# Preventive strategies for hypercoagulation in Cushing’s syndrome: when and how

**DOI:** 10.1186/s12959-023-00515-1

**Published:** 2023-07-03

**Authors:** Valentim Lopes, Olinda Marques, Adriana De Sousa Lages

**Affiliations:** 1 Endocrinology Department of Hospital of Braga, Braga, Portugal; 2 Faculty of Medicine, University of Coimbra, Coimbra, Portugal

**Keywords:** Thromboprophylaxis, Hypercortisolism, Cushing’s syndrome, Low molecular weight heparin

## Abstract

**Purpose:**

The endogenous hypercortisolism that characterizes Cushing’s syndrome (CS) is associated with a state of hypercoagulability that significantly increases the risk of thromboembolic disease, especially, venous events. Despite this certainty, there is no consensus on the best thromboprophylaxis strategy (TPS) for these patients. Our aim was to summarize the published data about different thromboprophylaxis strategies, and to review available clinical tools assisting thromboprophylaxis decision making.

**Methods:**

Narrative review of thromboprophylaxis strategies in patients with Cushing’s syndrome. A search was carried out on PubMed, Scopus and EBSCO until November 14th, 2022, and articles were selected based on their relevance and excluded in case of redundant content.

**Results:**

Literature is scarce regarding thromboprophylaxis strategies to be adopted in the context of endogenous hypercortisolism, most often being a case-by-case decision according to the centre expertise. Only three retrospective studies, with a small number of patients enrolled, evaluated the use of hypocoagulation for the thromboprophylaxis of patients with CS in the post-operative period of transsphenoidal surgery and/or adrenalectomy, but all of them with favourable results. The use of low molecular weight heparin is the most frequent option as TPS in CS context. There are numerous venous thromboembolism risk assessment scores validated for different medical purposes, but just one specifically developed for CS, that must be validated to ensure solid recommendations in this context. The use of preoperative medical therapy is not routinely recommended to decrease the risk of postoperative venous thromboembolic events. The peak of venous thromboembolic events occurs in the first three months post-surgery.

**Conclusion:**

The need to hypocoagulate CS patients, mainly in the post-operative period of a transsphenoidal surgery or an adrenalectomy, is undoubtable, especially in patients with an elevated risk of venous thromboembolic events, but the precise duration and the hypocoagulation regimen to institute is yet to be determined with prospective studies.

## Introduction

Cushing’s syndrome (CS) is associated with an eighteen-fold higher risk of venous thromboembolic events (VTE) compared with the general population [1]. Indeed, approximately 8% of patients with CS have had a VTE in the peri-diagnostic period [2–17]. Table 1 summarizes the risk of VTE (and also of cardiovascular events) reported in various studies that evaluated patients with CS. More importantly is that this state of hypercoagulability is present during the active phase of the disease and still persists in the postoperative period and even after achievement of biochemical remission [1, 18].

**Table 1:** Summary of studies reporting risk of venous thromboembolism and cardiovascular events in patients with Cushing’s syndrome

Author, year	Patients (*n*)	Risk of VTE	Risk of CV events
Huckhagel et al., 2023^2^	30	6.7%	-
Suarez et al., 2020^3^	208	9.8%	9%
Babic et al., 2019^4^	310	2.6%	-
Barbot et al., 2015^5^	78	3.8%	-
Koutroumpi et al., 2013^6^	58	14%	-
Van der Pas et al., 2012^7^	17	17.6%	-
Stuijver et al., 2011^8^	473	7.8%	-
Manetti et al., 2010^9^	40	7.5%	-
Kastelan et al., 2009^10^	33	9.1%	-
Sudhakar et al., 2004^11^	22	4.5%	-
Rees et al., 2002^12^	54	5.6%	-
Boscaro et al., 2002^13^	307	9.4%	-
Semple et al., 1999^14^	105	3.8%	-
Fahlbusch et al., 1986^15^	101	7.9%	-
Small et al., 1983^16^	53	11%	7.5%
Zanon et al., 1982^17^	15	13.3%	-
Total	1904	8.4%*	8.3%*

CD – Cushing’s disease, CS – Cushing syndrome, CV – cardiovascular, VTE – venous thromboembolism events

* mean

This hypercoagulable state is consequence of both quantitative and qualitative alterations in the haemostatic system induced by the cortisol excess.

In one hand, by the increase in plasma clotting factors - especially factor VIII and Von Willebrand factor (VWF), but also factors IX, X and XI -, the decrease in plasma tissue factor pathway inhibitor (TFPI) and the impairment of fibrinolysis – by the upregulation of the synthesis of plasminogen activator inhibitor type I (PAI-1). On another hand, the overexpression of abnormally high molecular weight VWF multimers capable of inducing spontaneous platelet aggregation contribute to a higher risk of thrombotic events.

Coagulation profiles in patients with CS are heterogeneously affected. The hemostatic abnormalities most consistently reported are shortening of activated partial thromboplastin time (aPTT) and increased thrombin generation [19–22]. Interestingly, increased VWF levels are not a constant feature reported in CS, depending on particular polymorphisms in the VWF gene promoter. As an example, haplotype 1 of VWF gene promoter confers a greater risk of VWF upregulation by cortisol and O blood group individuals have a 25% lower level of VWF than those with other ABO blood group, influencing data interpretation [19, 20].

This hypercoagulable state that characterizes CS is even higher when the patient undergoes a transsphenoidal surgery (TSS) or an (unilateral or bilateral) adrenalectomy to treat the hypercortisolism [6]. Babic B et al. demonstrated, in a retrospective study involving 4217 patients undergoing adrenalectomy for multiple causes, that the rate of post-operative VTE were higher in patients with CS (2.6% vs. 0.9%, *p* = 0.007) [4]. Stuijver et al., in a cohort of 473 patients (360 with ACTH-dependent pituitary CS and 113 with non-functioning pituitary adenoma) submitted to TSS, found a higher rate of VTE 3 months after surgery in the first group (3.4% vs. 0%, *p* = 0.01) [8].

So, the risk of VTE associated with Cushing’s syndrome is significant and is increased, especially in the post-operative period, approaching that of a major orthopaedic surgery. That was established by numerous studies that have accepted the challenge launched by the Endocrine Society in 2015, when, in the section of “Future Directions and Recommended Research” of the Clinical Practice Guideline about the treatment of Cushing’s syndrome, Nieman LK *et al* stated the need to «evaluate the utility of venous thromboembolism prophylaxis before and after remission”, but the evaluation and comparativeness of different regimens of thromboprophylaxis in patients with CS by large prospective studies still does not exist and the data that we actually have regarding this issue result mainly from few small retrospective studies and from expert consensus [23]. Indeed, a recent report that aimed to map the current clinical practice for thromboprophylaxis management in patients with CS across reference centres (RC) of the European Reference Network on Rare Endocrine Conditions (Endo-ERN) revealed that the majority of the RC provided thromboprophylaxis to patients with CS, but only one centre had a standardized thromboprophylaxis protocol [24].

Our aim was to summarize the published data about different thromboprophylaxis strategies, and to review available clinical tools assisting thromboprophylaxis decision making.

## Materials and methods

We present a narrative review with the intent to address the following question: in patients with Cushing’s syndrome, is the implementation of thromboprophylaxis strategies effective in reducing the risk of VTE? Three electronic databases (PubMed, Scopus and EBSCO) were searched in November 2022 to identify potentially relevant articles. Randomized controlled trials and cohort studies assessing thromboprophylaxis strategies in patients with CS were elegible. All articles were read by two independent authors and selected based on their relevance and excluded in case of redundant or inadequate (for the aim of this review) content.

Firstly, for the contextualization of the issue, in other words to explain the necessity to implement a thromboprophylaxis strategy in patients with CS, we searched articles using the terms “Cushing syndrome” [MeSH Terms] OR “Cushing disease” [MeSH Terms] OR “hypercortisolism” [MeSH Term] AND “coagulation” [MeSH Term] OR “hypercoagulability” [MeSH Terms]. Secondly, with the intention to find articles that could bring us some light about different thromboprophylaxis strategies implemented in these patients, and also their benefits and risks, with carried out the research with the terms “Cushing syndrome” [MeSH Terms] OR “Cushing disease” [MeSH Terms] OR “hypercortisolism” [MeSH Term] AND “anticoagulant agents” [MeSH Terms].

## Results

### Thromboprophylaxis strategies

Only three retrospective studies (Table 2), with a small number of patients enrolled, evaluated the use of hypocoagulation for the thromboprophylaxis of patients with CS in the post-operative period of transsphenoidal surgery and/or adrenalectomy [25].

**Table 2:** Clinical studies regarding thromboprophylaxis strategies implemented in the context of endogenous hypercortisolism

Author, year	Etiology of CS	Patients (*n*)	Thromboprophylaxis strategy	Incidence of postoperative VTE
Boscaro M et al., 2002^13^	61% CD 19% ACA 8% ACC	75	No anticoagulation	20%
	65% CD 15% ACA 7% ACC	232	Non-fractioned heparin followed by warfarin	6%
Barbot M et al., 2015^5^	100% CD	34	LMWH during 14 days plus universal GC coverage	8.8%
		44	LMWH during 30 days plus elastic compression stockings and early mobilization	0
Suarez MG et al., 2020^3^	89% CD 7% AC 4% EC	147	No anticoagulation	23%
		50	LMWH	10%

ACA – adrenocortical adenoma, ACC – adrenocortical carcinoma, CD – Cushing’s disease, CS – Cushing syndrome, EC – ectopic Cushing syndrome, GC – glucocorticoid, LMWH – Low-molecular weight heparin, VTE – venous thromboembolism events

The first one was done by Boscaro M et al. in 2002 [13]. They compared two groups of patients with CS of different aetiologies (Cushing’s disease, adrenal adenoma and carcinoma or ectopic secretion of ACTH) undergoing surgery to treat the hypercortisolism: the first group, with 75 patients, did not received any type of hypocoagulation, and the second group, with 232 patient, received hypocoagulation with non-fractioned heparin during 22 days followed by 4 months of warfarin therapy (or until the remission of the disease). The incidence of VTE was, respectively, 20 and 6% (*p* < 0.001), without any significant haemorrhagic event in the group of patients that received hypocoagulation strategy.

The second one was conducted in 2015 by Barbot M et al. [5]. They compared two groups of patients with a diagnosis of Cushing’s disease submitted to TSS. 34 patients were hypocoagulated with enoxaparin (4,000 U) or nadroparin (3,800 U) until the discharge or until 14 days after the surgery (plus universal glucocorticoid coverage) and 44 patients received enoxaparin during thirty days associated with the use of graduated elastic compression stockings and were instructed to mobilize precociously (plus glucocorticoid coverage only if the cortisol level 48 h after the surgery was < 270 nmol/L or 9.8 µg/dL). The incidence rate of VTE was 8.8% in the first group (with 2.9% of these VTE being a fatal event) and none VTE occurred in the second group (*p* = 0.081). No bleeding complications were observed during the follow-up in either group.

The last study to evaluate a thromboprophylaxis strategy in patients with CS was conducted in 2020 by Suarez MG et al. [3]. It was a single-centre, retrospective study that enrolled 208 patients with CS. 197 of these individuals did a surgery to treat the hypercortisolism (bilateral adrenalectomy only, TSS plus bilateral adrenalectomy, TSS plus unilateral adrenalectomy, TSS only or unilateral adrenalectomy only). 50 patients received prophylactic hypocoagulation with enoxaparin, of whom only 5 had a TEV (10%) and, in the other hand, of the 147 patients that didn’t receive hypocoagulation, 34 had a TEV (23%).

Aspirin, a nonsteroidal anti-inflammatory drug with anti-platelet activity, was also used with a thromboprophylaxis intent during 6 weeks from day 1 post-operatively in patients with CD submitted to transsphenoidal surgery in an american department of neurologic surgery. Although the authors mentioned a reduction in the rate of DVT risk after the implementation of this strategy, there is no factual data about it, namely the number of DVT episodes before and after the use of aspirin, the dose used, the complications that resulted from this strategy, etc. [26].

Regarding the use of direct oral anticoagulants (DOACs), as apixaban, rivaroxaban, dabigatran and edoxaban, that are now indicated and recommended for extended prophylaxis in various orthopaedic surgeries, no study investigated its efficacy and safety in CS context. Considering its simplicity and successful use in other high-risk contexts, it may become a valid alternative also in outpatients with CS at higher risk of VTE [24].

It is also important to highlight non-pharmacological measures. Most centres use compression stockings until hospital discharge and early mobilization as a universal combination strategy for CS patients. Other mechanical strategies as intermittent pneumatic leg compression and regular foot dorsiflexion exercises are also used with good results in some reference centres [24].

### Duration of thromboprophylaxis

The duration of the thromboprophylaxis after the diagnosis and, specially, after biochemical remission of the disease is another question of debate.

Numerous studies have demonstrated that the state of hypercoagulability persists during some time after the accomplishment of the remission (normalization of the cortisolemia), perhaps because of the maintenance of the abdominal obesity and the use of supraphysiological glucocorticoid replacement doses in the postoperative period [3, 7, 9, 13]. This lag time between the remission of the CS and the normalization of the biochemical parameters of coagulation is variable.

Manetti L et al. demonstrated that, one year after the TSS, the hypercoagulable state, although some improvement in the haemostatic parameters was observed, still persisted [9].

Ferrante E et al. also demonstrated that the hypercoagulability persisted six months after the surgery (TSS or adrenalectomy), but five years after it was completely reverted in those patients that accomplished remission of the disease (defined by the authors as adrenal insufficiency or normal pituitary/adrenal axis function in the presence of normal urinary free cortisol levels, normal value of late-night salivary cortisol and positive response to low-dose dexamethasone suppression test) [27].

On the other hand, Kastelan D et al., in a prospective case-control study that enrolled 36 patients (18 with CS and 18 without any disease) demonstrated that, 6 months after the remission of the disease (accomplished by TSS or adrenalectomy and herein defined by the authors as normal urinary free cortisol levels, cortisol diurnal rhythm and cortisol suppression after 1 mg overnight dexamethasone), the risk of VTE in the two groups was similar [10].

Nevertheless, there is a certainty: the risk of VTE is highest in the first 3 months post-surgery despite some temporal heterogeneity between studies.

Stuijver DJF et al. revealed that the majority of the VTE events occurred between the first- and second-week post-surgery [8]. In the study conducted by St-Jean M et al. and Suarez MG et al., 50 and 40%, respectively, of the events occurred in the first 2 months after the surgery [3, 25]. And Boscaro et al. revealed that 62% of the VTE events occurred in the first three months post-surgery [13].

Interestingly, this peak of the risk of VTE in the postoperative period seems not only to be related with the surgery itself but also to an acute cortisol drop that occurs in the postoperative period and that it is hypothesized to induce a rebound pro-inflammatory (and thus pro-coagulant) response. This fact may be mediated by an increase in the number of lymphocytes secondary to the loss of Th1 cell suppression, with a consequent increase in the levels of different cytokines such as IFN-γ, IL-2 and TNF-β [1, 4, 28–30]. In the same line, a more aggressive decrease in cortisol levels in patients undergoing bilateral adrenalectomy may also be associated with a higher and more prolonged risk of postoperative VTE [3].

It should also be noted that due to various comorbidities such as osteoporosis and myopathy, patients with CS are themselves subject to less mobilization after surgery, which, in a way, may also partially influence the risk of thrombosis in the postoperative period.

van Haalen et al. demonstrated that 48% of the RC of the Endo-ERN initiated the thromboprophylaxis when the diagnosis of CS was made, 26% on the day before/of the surgery, 17% preoperatively, 13% postoperatively and 9% depending on the presentation (the responses were not mutually exclusive).

Regarding the time for abrogation of thromboprophylaxis, in the RC in which it was standardized (35% of the centres), 38% centres stopped at 1 month postoperatively, 25% between 2 and 4 weeks postoperatively and the remaining 37% equally distributed for “between 1 week before and 2 weeks after surgery”, “between 4 and 6 days postoperatively” and “at 3 months postoperatively”. In the other hand, in the remaining 65% of RC in which the abrogation of thromboprophylaxis was individualized, 60% stopped the hypocoagulation as soon as the patient was no longer immobile, 40% upon achievement of remission, 27% depending on patient status and 7% based upon haemostatic parameters (the responses were again not mutually exclusive). Summarizing the available data, stopping thromboprophylaxis is mainly based on individual characteristics rather than standardized treatment duration but depended most frequently on the mobility factor of the patient [24].

Some authors propose the decision of discontinuation based upon haemostatic parameters, but the use of coagulation parameters alone to determine risk has not been firmly established. Additionally, standardized evaluation of inherited risk factors (as Factor V Leiden, prothrombin gene 20,210 A variants or genotype GCAG/GCAG of the VWF gene promoter region) may be not routinely available in the majority of centres.

### The usefulness of preoperative medical therapy and its impact on the strategy of hypocoagulation

Another question that was investigated was if the medical therapy to treat endogenous hypercortisolism preoperatively (PMT) could significantly diminish the risk of VTE in the postoperative period.

Stuijver et al. demonstrated that the risk of VTE 3 months after surgery was 2.5% (95% CI 1.2–5.1%) in the group of patients that received preoperative medical therapy (PMT) vs. 7.2% (95% CI 3.1–15.9%) in the group that did not receive any target therapy. However, the authors did not mention which PMT options were chosen for each patient, limiting the sub-analysis of the efficacy of the different pharmacological agents actually available to treat CS [8].

On the other hand, data from a large European registry (ERCUSYN) did not show any difference in the prevalence of postsurgical thromboembolism between patients with CS who received and did not receive PMT [31]. Also, van der Pas et al., in a study that enrolled 17 patients with CD treated with pasireotide during 1 month (with the addition of cabergoline in case of persistent hypercortisolism), demonstrated that, 12 weeks after the achievement of biochemical remission of CS, despite the decrease in the plasma levels of antithrombin and thrombin-activatable fibrinolysis inhibitor, there was no improvement in other coagulation indices, namely a reduction in the level of coagulation factors [7].

Overall, an association between preoperative medical treatment and reduction of VTE risk in patients with CS remains controversial and taking in consideration the lack of effect of medical therapy on changing either clotting or anticoagulant factors during therapy, current evidence does not support the use of medical therapy focusing on TVE risk mitigation.

### Clinical factors to be considered

Although active CS is associated with a moderate to high risk of VTE, no significant correlation between the severity of hypercortisolism and haemostatic abnormalities was demonstrated [1]. Nevertheless, most of the reference centres in Europe use clinical criteria for the decision to initiate or not thromboprophylaxis, namely VTE risk factors (as previous VTE, severity of hypercortisolism and limitation of mobility) and traditional cardiovascular disease risk factors (as older age, current smoking and presence of other neoplastic disease) [24]. Naturally, patients with known hereditary thrombophilia (for example Factor V Leiden and Prothrombin gene 20,210 A variants) were hypocoagulated with no doubt. CS subtype was another relevant clinical factor influencing the initiation of thromboprophylaxis. Patients with ectopic ACTH/CRH syndrome or malignant adrenal CS were considered to have an additional malignancy-related risk of VTE, although the reason for the differences in VTE incidence in patients with different CS aetiologies is not entirely clear.

### Risk of VTE

There are numerous VTE risk assessment scores validated for different medical purposes, but just one specifically developed for CS [32–36]. Table 3 depicts the different scores actually available to calculate the individual risk of VTE.

**Table 3:** Different models to assess venous thromboembolism events risk

Score	When to use	Variable – point(s)	Interpretation
Modified Caprini VTE Risk Assessment^32^	Patients undergoing non-orthopedic surgery	Age – 1 to 3 Type of surgery – 1, 2 or 5 Medical history – 1,2,3 or 5 Physical signs – 1 Other risk factors – 1	0 (very low risk) – no thromboprophylaxis
			1–2 (low risk) – mechanical prophylaxis*
			3–4 (moderate risk) – LMWH + mechanical prophylaxis
			≥ 5 (high risk) – LMWH + mechanical prophylaxis
Geneva Risk Score^33^	Hospitalized medical patients	Diseases with increased VTE risk – 2 (each) Known hypercoagulable state – 2 Immobilization for ≥ 3 days – 1 Recent travel for > 6 h – 1 Age > 60 years – 1 Obesity – 1 Chronic venous insufficiency – 1 Pregnancy – 1 HT – 1 Dehydration – 1	< 3 (low risk) – no VTE prophylaxis indicated
			≥ 3 (high risk) – VTE prophylaxis indicated
Padua Prediction Score^34^	Hospitalized patients	Active cancer – 3 Previous VTE – 3 Reduced mobility – 3 Known thrombophilic condition – 3 Recent trauma/surgery – 2 ≥ 70 years – 1 Heart/Respiratory failure – 1 Acute MI/ischemic stroke – 1 Acute infection/rheumatologic disorder – 1 Obesity – 1 Ongoing HT – 1	< 4 (low risk) – pharmacologic prophylaxis is NOT indicated. Consider using mechanical prophylaxis
			≥ 4 (high risk) – pharmacologic prophylaxis is indicated. If high risk of bleeding, use mechanical prophylaxis
IMPROVEDD Risk Score^35^	Hospitalized patients	Previous VTE – 3 Known thrombophilia – 2 Lower-limb paralysis – 2 Current cancer – 2 Immobilized ≥ 7 days – 1 ICU/CCU stay – 1 Age > 60 years – 1 D-dimer ≥ 2xULN – 2	Calculates the 42 and 77-day VTE risk, but doesn´t make any recommendation regarding the need of VTE prophylaxis
CS-VTE Score^36^	Patients with active CS	Age ≥ 69 years – 2 Reduced mobility – 2 Acute severe infection – 1 Previous CV events – 1 Midnight plasma cortisol > 3.15xULN – 1 Shortened APTT – 1	Stratify the VTE risk (2 points - low risk, 3 points - moderate risk, 4 points -high risk, and ≥ 5 points - very high risk), but doesn’t make any recommendation regarding the need of VTE prophylaxis

APTT – activated partial thromboplastin time, CCU – coronary care unit, CS – Cushing’s syndrome, HT – hormonal therapy, ICU – Intensive Care Unit, LMWH – low-molecular weight heparin, ULN – upper limit of normality, VTE – venous thromboembolism events, *intermittent pneumatic leg compression or elastic stockings

The modified Caprini Score is a scoring tool used to quantify and categorize a patient’s risk for venous thromboembolism and have been developed to evaluate the risk of perioperative VTE in most general surgical interventions. Based on this score, a vast majority of CS patients undergoing surgery will be at moderate, and some at high risk of VTE. Within this line, if we consider CS to be an acquired thrombophilic state, all patients would be at high risk of VTE [32].

The only tool specifically designed for patients with CS is the CS-VTE Score published in 2016, which included 176 patients with active CS and evaluated both clinical and biochemical parameters (as shortened aPTT). Although promising, this model has not yet been validated in other studies and only categorizes patients according to their VTE risk, with no specific recommendations regarding the best strategy to adopt in each case [36].

### Risk of haemorrhage

Although endogenous hypercortisolism is traditionally associated to bleeding tendency, some authors hypothesize that this fact may be only theoretical. In fact, no increased bleeding complications were found in patients with CS undergoing laparoscopic adrenalectomy and bruising or poor wound healing that characterizes the clinical picture of CS are thought to be the result of alterations in synthesis of skin components rather than specific coagulation disorders [24].

But, from the clinical point of view, treatment with anticoagulation agents is naturally accompanied by an increased risk of major bleeding, so this concern must be present before taking the final decision to hypocoagulate or not the patient. There are numerous haemorrhagic risk assessment scores to access the risk-benefit of hypocoagulation, but all of them are validated for patients with atrial fibrillation and none for other purposes, namely for patients with active endogenous hypercortisolism. Table 4 outlines the different scores available, of which HAS-BLED is the most used [37–40].

**Table 4:** Different models to assess patients’ haemorrhagic risk

Score	When to use	Variable – point(s)	Interpretation
HAS-BLED Score^37^	Patients with atrial fibrillation	Hypertension – 1 Abnormal liver/renal function – 1/2 Stroke history – 1 Bleeding predisposition – 1 Labile INR – 1 Elderly (age > 65 years) – 1 Drug/alcohol usage − 1/2	≤ 1 (low risk) – anticoagulation should be considered
			2 (moderate risk) – anticoagulation can be considered
			3–5 (high risk) and > 5 (very high risk) – alternatives to anticoagulation should be considered
HEMORR_2_HAGES Score^38^	Elderly patients with atrial fibrillation	Hepatic or renal disease – 1 Ethanol abuse – 1 Malignancy – 1 Older (age > 75 years) – 1 Reduced platelet count or function – 1 Rebleeding (prior bleed) – 2 Hypertension (uncontrolled) – 1 Anemia – 1 Genetic factors (CYP2C9 SNP) – 1 Excessive fall risk – 1 Stroke – 1	≤ 1 (low risk) – consider anticoagulation if clinically indicated
			2–3 (intermediate risk) – consider alternatives to anticoagulation unless strong indications for it exists
			≥ 4 (high risk) – alternative options should often be considered
ATRIA Bleeding Risk Score^39^	Patients in whom warfarin anticoagulation is being considered	Anemia – 3 Severe renal disease/Dialysis – 3 Age ≥ 75 years – 2 Prior hemorrhage – 1 Hypertension – 1	< 4 points (low risk) – reasonable to start warfarin
			4 points (intermediate risk) – alternatives to warfarin therapy can be considered
			> 4 points (high risk) – alternatives to warfarin should be strongly considered
ORBIT Score^40^	Patients with atrial fibrillation	Anemia – 2 Age > 74 years – 1 Bleeding history – 1 GFR < 60 mL/min/1.73 m^2^ – 1 Treatment with antiplatelet agents – 1	≤ 2 (low risk) 3 (medium risk) 4–7 (high risk) Doesn´t make any recommendation regarding the use or not of hypocoagulation

GFR – glomerular filtration rate, INR – international normalized ratio (prothrombin time), SNP – single-nucleotide polymorphism

## Discussion

The three studies that demonstrated a reduction in VTE when using postoperative thromboprophylaxis provide a first step of evidence in the use of hypocoagulation for thromboprophylaxis in patients with CS. However, it is important to point out some important biases that limit their generalization to clinical practice. Namely: retrospective methodology, small number of patients enrolled, and great heterogeneity.

Given the vast amount of data collected from studies that have addressed this issue over the past twenty years, two recently published expert consensus have recommended the use of hypocoagulation for thromboprophylaxis of patients with CS with a frankly elevated risk of VTE, pointing out some clinical criteria of major relevance, namely history of VTE, thrombophilic states, severe hypercortisolism, use of estrogen/testosterone, prolonged preoperative and postoperative periods, high postoperative cortisol levels and glucocorticoid overreplacement in the postoperative period. Furthermore, an ESE Clinical Practice Guideline published in 2021 also recommends treating pregnant women with active Cushing’s disease with prophylactic anticoagulation [41].

The risk of VTE, and consequently the need for thromboprophylaxis, can be calculated based on various risk prediction models, of which the *Caprini* VTE Score, although not specifically validated to CS, seems to be, for patients with CS in the post-operative period, the most suitable, especially if combined with the CS-VTE Score calculation, a risk assessment tool specifically designed for patients with active CS patients.

Simultaneously, the expert consensus recommend that the clinician should also evaluate the haemorrhagic risk of each patient before taking the final decision to hypocoagulate or not the patient. That risk can be assessed by multiple scores, namely HAS-BLED, the most used and validated hemorrhagic risk score. Although specifically designed for patients with atrial fibrillation, it could help clinicians to identify some factors that, in addition to the cutaneous fragility (and consequently easy bruising) characteristic of CS, put this patients at a higher risk of bleeding if pharmacologic thromboprophylaxis is initiated.

Several authors suggest starting thromboprophylaxis 24 to 48 h after surgery and continuing for at least 2 to 6 weeks, although it can be extended to 2 to 3 months in patients with the highest thrombotic risk, particularly patients who have undergone a bilateral adrenalectomy. They also state that it is possible, especially in patients with a frankly elevated risk of VTE, to initiate the thromboprophylaxis immediately after the diagnosis and, in this case, the anticoagulant should be held before the surgery (timing adjusted based on the anticoagulation regimen chosen) [28, 42, 43].

The thromboprophylaxis agents most used in the postoperative period and approved by the European Medicines Agency (EMA) include the low-molecular weight heparin (LMWH) enoxaparin, the factor Xa inhibitor, fondaparinux, and the direct oral anticoagulants (DOAC) rivaroxaban, apixaban and dabigatran (Table 5) [44]. LMWH is the first-choice anticoagulant drug used for patients with CS in all the RC of the Endo-ERN in which thromboprophylaxis is routinely implemented [23]. There isn’t, to date, any study that have evaluated the role of DOAC in patients with CS, but a multicentre controlled study that randomized 206 patients with CD submitted to transsphenoidal surgery into 2 groups (mechanical prevention *versus* mechanical prevention plus LMWH followed by rivaroxaban) is ongoing an can bring us new insights [45]. So, based on the data currently available and the vast experience with LMWH, with a good tolerability and risk-benefit profile, it seems to be, actually, the preferred drug to use in the thromboprophylaxis of patients with CS, even in pregnancy [41].

**Table 5:** Thromboprophylaxis agents approved by the EMA

Agent class	Example	Dose	Comments	Dose adjustment
LMWH	Enoxaparin	40 mg sc od	Approved for patients submitted to orthopedic or general surgery and for non-surgical patients with an acute disease and reduced mobility	If GFR < 30 mL/min/1.73 m^2^, reduce to 20 mg sc od
			Stop 24 h pre-op.	
			Start 6 h post-op.	
Factor Xa inhibitor	Fondaparinux	2.5 mg sc od	Approved for adults submitted to hip or knee surgery and in adults at high risk of VTE who are having abdominal surgery or who are forced to stay in bed	For patients with “kidney problems”, it may not be suitable or the dose of 1.5 mg may be used
			Stop 36-42 h pre-op.	
			Start 6 h post-op.	
			An alternative to enoxaparin in case of heparin-induced thrombocytopenia	
DOAC*	Rivaroxaban	10 mg po od	Approved for adult patients submitted to knee or hip arthroplasty	If GFR < 15 mL/min/1.73m^2^, its use is not recommended
			Stop 48 h pre-op.	
			Start 6-10 h post-op.	
	Apixaban	2.5 mg po bid	Approved for adult patients submitted to knee or hip arthroplasty	If GFR < 15 mL/min/1.73m^2^, its use is not recommended
			Stop 46 h pre-op.	
			Start 12-24 h post-op.	
	Dabigatran	110 mg po in the day of the surgery 220 mg po od thereafter	Approved for adult patients submitted to knee or hip arthroplasty	If GFR 30–50 mL/min/1.73 m^2^ and age ≥ 75 years, 75 mg po in the day of the surgery and 150 mg po od thereafter
			Stop 48, 72 and 96 h pre-op. if GFR ≥ 80, 50–80 and 30–50 mL/min/1.73 m^2^, respectively	
			Start 1-4 h post-op. (the dose of 110 mg)	

bid – twice daily, GFR – glomerular filtration rate, od – once day, po – *per os*, sc – subcutaneous

*Ketoconazole may increase levels of rivaroxaban, apixaban and dabigatran, so it may be prudent to avoid the combination or to consider a therapy modification

It is also important to mention that this pharmacologic thromboprophylaxis should be used in addition to the mechanical thromboprophylaxis, e.g., intermittent pneumatic compression stockings or elastic stockings, to augment the efficacy of the thromboprophylaxis strategy.

With respect to the use of PMT to reduce the risk of post-operative VTE, given its unclear benefit and potential adverse effects, it is not routinely recommended. Nevertheless, it should be offered on an individual basis, especially if surgery is delayed or if hypercortisolism is markedly severe [28].

**Fig. 1:**
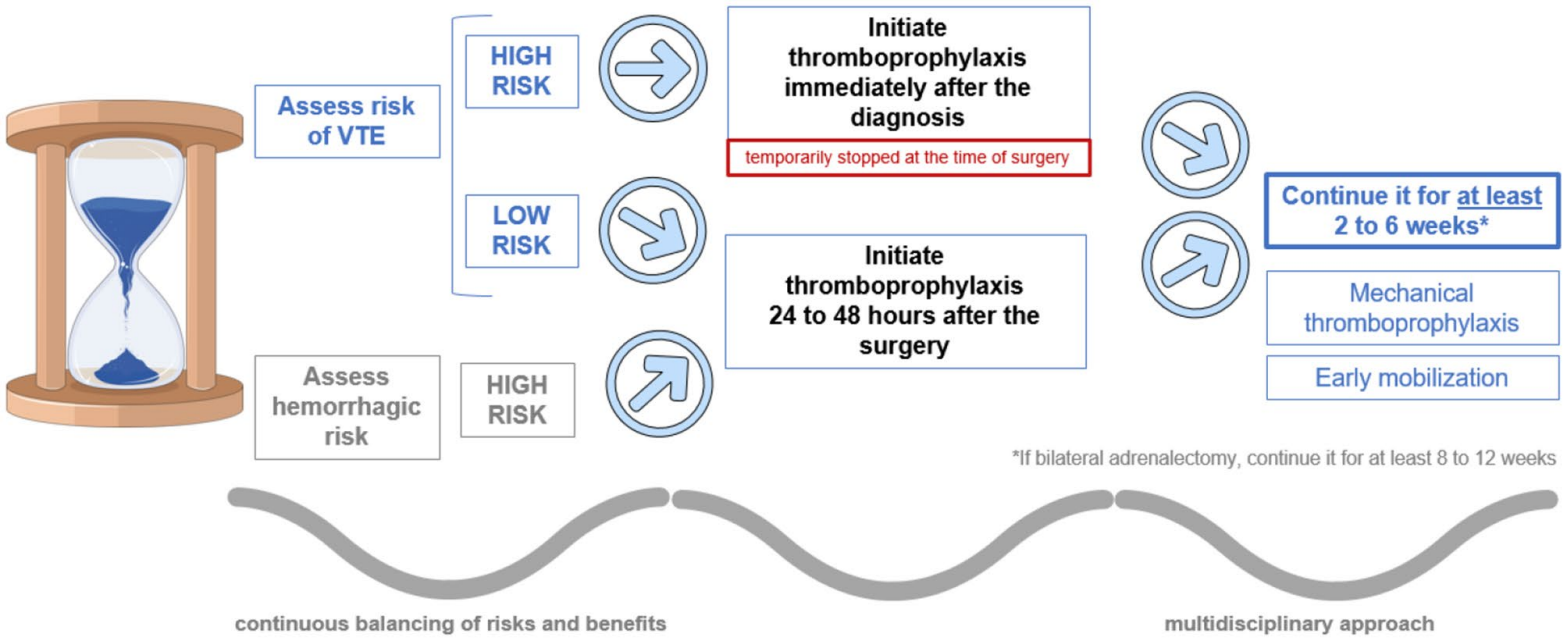
Proposal of thromboprophylaxis regimen in patients with CS

In conclusion, thromboprophylaxis strategy is not about a single, simple decision to initiate or not hypocoagulation drugs. It is all about the continuous balancing of risks and benefits, which must always be individualized, in the pre and postoperative period, for each patient who presents with CS. Nevertheless, based on the literature reviewed here, we can state a general recommendation about the adequate strategy of thromboprophylaxis to implement in patients with CS (Fig. 1).

## Data Availability

Not applicable.
